# Mechanism of Androgen-Independent Stromal Proliferation in Benign Prostatic Hyperplasia

**DOI:** 10.3390/ijms241411634

**Published:** 2023-07-19

**Authors:** Junya Hata, Yuki Harigane, Kanako Matsuoka, Hidenori Akaihata, Kei Yaginuma, Satoru Meguro, Seiji Hoshi, Yuichi Sato, Soichiro Ogawa, Motohide Uemura, Yoshiyuki Kojima

**Affiliations:** Department of Urology, Fukushima Medical University School of Medicine, 1 Hikarigaoka, Fukushima 9601295, Japan; uroharry@fmu.ac.jp (Y.H.); kanaco@fmu.ac.jp (K.M.); hakai@fmu.ac.jp (H.A.); uro-yagi@fmu.ac.jp (K.Y.); s-meguro@fmu.ac.jp (S.M.); uro-hoshi@fmu.ac.jp (S.H.); ysato@fmu.ac.jp (Y.S.); soh@fmu.ac.jp (S.O.); muemura@fmu.ac.jp (M.U.); ykojima@fmu.ac.jp (Y.K.)

**Keywords:** benign prostatic hyperplasia, stromal proliferation, androgen independent, autoimmune reaction

## Abstract

Benign prostatic hyperplasia (BPH) is a chronic proliferative disease showing stromal-dominant proliferation. However, the detailed proliferation mechanism has remained unclear. Although aging and androgen have been reported as definitive risk factors for BPH, recent studies have focused on the involvement of androgen-independent factors. Androgen-independent factors include ischemia, oxidative stress, metabolic syndrome, infection, autoimmune reactions, and inflammation, with inflammation in BPH tissues playing a central role in the BPH proliferative process. Inflammation in BPH tissues by various factors finally leads to tissue remodeling and stromal proliferation through the wound healing process of the prostate. To elucidate the proliferative mechanism of BPH, a study using whole-genome gene expression analysis in a stromal-dominant BPH rat model was performed and showed that immune response-related pathways and complement classical pathways are activated. Furthermore, expression analysis using this BPH rat model showed that the autoimmune reaction triggered complement pathway activation in the proliferative process of BPH. BPH is a multifactorial disease, and understanding the role of androgen-independent factors including immune responses contributes to elucidating the pathogenesis of BPH. Androgen-independent factors may lead to new therapeutic targets for BPH, and further development of this research is expected.

## 1. Introduction

Benign prostatic hyperplasia (BPH) is a chronic proliferative disease that may be defined as prostate glandular epithelial enlargement secondary to epithelial and stromal hyperproliferation, with a predominance of stromal cells [1]. BPH is a progressive condition of aging men that McNeal characterized as a selective benign overgrowth within the transition-periurethral zone (TPZ) proximal to the verumontanum, which can clinically result in bladder outlet obstruction and various lower urinary tract symptoms (LUTS) [2,3,4,5,6]. Beginning at age 40 years, the prevalence of BPH increases with aging. Approximately fifty percent of males can exhibit BPH symptoms by 51–60 years [7]. The prevalence of BPH was seventy percent in males by 70 years, and increases to eighty percent by 85 years [8]. The occurrence and development of BPH are closely related to age.

The human prostatic gland is composed of secretory epithelium arranged in the gland within a fibromuscular stroma composed of smooth muscle. Understanding prostate development clarifies some of the hyperplastic changes observed in BPH, since this disease has been seen historically as a type of embryonic reawakening process [4,9]. Prostatic embryonic development, subsequent pubertal and adult growth, and homeostatic maintenance are strongly regulated through androgen-dependent reciprocal paracrine interactions between stromal and epithelial components (stromal-epithelial interaction) [10]. Cell proliferation is greatly increased in BPH compared to normal prostate tissue; epithelial proliferation was 9-fold higher, while stromal proliferation was 37-fold higher [11], suggesting that BPH is a disease that has stromal-dominant proliferation. Prostate epithelial proliferation results in enlargement of glandular nodules, while stromal proliferation produces a more diffuse hyperplasia with increased extracellular matrix production including collagen type I [12]. However, the specific mechanisms that promote prostatic enlargement, as well as the pathological changes leading to the BPH phenotype, have remained unclear.

**Figure 1:**
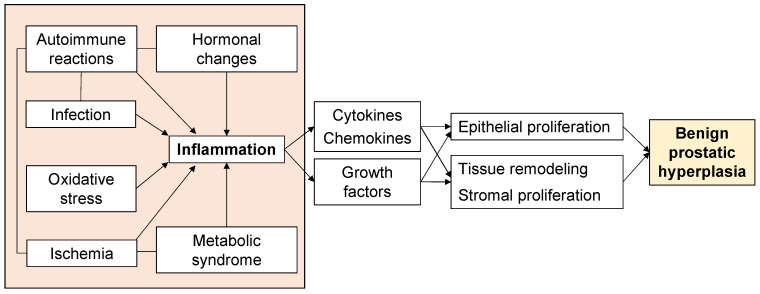
Factors associated with the proliferative process of BPH.

Aging and androgens are necessary for the development of BPH, but the pathogenesis of BPH is still largely unresolved [13]. Although androgen levels in men generally decrease with age, there is a paradox that prostate weight increases in BPH patients [14]. On the other hand, clinical practice has proven that a 5α-reductase inhibitor (5AR-I) can effectively reduce the level of dihydrotestosterone (DHT) in prostate tissue and reduce the risk of BPH progression, but 10% of patients still have clinical progression [15,16]. This shows that androgens are not the only factors that cause BPH. Androgen-independent factors include ischemia, oxidative stress, metabolic syndrome, infection, autoimmune reactions, and inflammation (Figure 1), and these interact with each other to form more complicated pathophysiology. In particular, inflammation has received much attention, and various studies are underway. Therefore, to elucidate the pathogenesis of BPH, an approach that focuses on androgen-independent factors is required.

This review focuses on the role of androgen-independent factors in stromal proliferation of BPH and the potential for novel therapeutic agents targeting these factors.

## 2. Histological Features of BPH

BPH is characterized as a chronic, progressive, but discontinuous hyperplasia of both glandular epithelial and stromal components leading to prostatic enlargement and clinical symptoms [9,17]. Franks and McNeal emphasized the idea that BPH is one of the nodular diseases, and that BPH nodules have several types including stromal, fibromuscular, muscular, fibroadenomatous, and fibromyoadenomatous [9,18]. The stromal components of BPH also contain fibroblasts, myofibroblasts, blood vessels, nerves, and inflammatory cells. Each of these epithelial and stromal components have the possibility of being involved in the development of BPH. Histological BPH can be defined as epithelial and stromal proliferation in the prostate transition zone associated with tissue remodeling, which involves epithelial tissue and fibromuscular matrix, with a certain androgen dependence [19,20,21]. Compared with normal prostatic tissue, the balance between growth and apoptosis of stromal cells in hyperplastic nodules is lost, finally leading to an increase in stromal volume.

McNeal also reported that the adult stromal nodule had re-acquired embryonic activity and was inducing the formation of a glandular duct that was invading into the nearby stromal nodule. This concept was based on the demonstration that embryonic prostatic development is induced by urogenital sinus mesenchyme (UGM), as the embryonic reawakening theory [22].

## 3. Stromal-Epithelial Interaction in BPH

The effect of UGM on prostatic development is one of the most basic developmental mechanisms, namely the stromal-epithelial interaction. The organs of the male and female urogenital tracts develop through stromal-epithelial interactions that are in sequence regulated by steroid hormones acting through mesenchymal and epithelial steroid receptors [23]. The importance of the stromal component in prostate proliferation has been described. In classic tissue recombination experiments, androgen signaling was reported to be required in the stroma, although it is dispensable in the epithelium for the induction of prostatic tissues [24]. In addition, mosaic ablation of the transforming growth factor (TGF) β type II receptor in a subset of fibroblasts was clarified to result in epithelial neoplasia [25,26]. Moreover, overexpression of fibroblast growth factor 10 by UGM promotes the growth of cancer in adult dissociated prostate epithelial cells grafted under the kidney capsule [27]. There are the interactions between the UGM and urogenital epithelia (UGE) in proliferative process of prostate. The UGM specifies prostatic epithelial identity and induces epithelial invading and budding, and the developing prostatic epithelium likewise induces smooth muscle differentiation and developing a pattern of UGM [28,29]. In transplantation experiments in which UGM alone is transplanted under the kidney capsule of male nude mice, a small amount of smooth muscle differentiated in the grafts [29]. On the other hand, tissue recombinants, indicating a mixture of two cells to evaluate their interaction with each other, composed of UGM and UGE induces the formation of prostatic ducts with epithelial cells surrounded by smooth muscle cells [30]. Importantly, smooth muscle cells can be specified in the UGM not only by UGE, but also by epithelium from adult prostate, presenting common inductive signals across epithelial types and stages [31]. The signaling by interaction between the epithelium and the stroma is not only crucial for prostate development, but it also continues to play key roles in maintaining prostate homeostasis in the adult prostate. These results demonstrate the importance of the stroma and stromal-epithelial interaction in the BPH proliferative process.

Several studies have reported the effectiveness of treatments targeting the stromal-epithelial interaction in BPH. One current study aims to elucidate the potential roles of bone morphogenic protein (BMP) 5 and correlated signaling pathways for the epithelial-mesenchymal transition (EMT) in BPH. This study reported that BMP5 was upregulated in human and rat hyperplastic prostate tissues and localized both in the epithelial and stromal area of prostate tissues. Overexpression of BMP5 enhanced cell proliferation and the EMT process through phosphorylation of small mother against decapentaplegic (Smad) 1/5/8, while selective knockdown of BMP5 induced cell cycle arrest at the G0/G1 phase and inhibited the EMT process. Moreover, the agonist and antagonist of BMP/Smad signaling pathway inverted the effects of BMP5 silence and overexpression, describing that BMP5 regulated cell proliferation and the EMT process through the BMP/Smad signaling pathway, which might contribute to the development of BPH [32].

Hedgehog (HH) signaling is reported to be a master regulator in numerous developmental processes [33]. HH signaling regulates ductal morphogenesis through stromal-epithelial interactions during prostate development [33]. In the adult prostate, HH signaling is active in the stromal component, and HH-responding cells show properties of stromal stem cells. The roles of HH signaling in prostate development have been previously reported by the Bushman group and others [34,35,36].

## 4. Stromal Proliferation in BPH

The stromal proliferation of BPH develops by two pathways, including androgen-dependent and androgen-independent pathways. The role of androgen in BPH development has been definitive. One study reported increased migration of macrophages and proliferation of prostate stromal cells in a co-culture trans well system. This result showed that targeting the androgen receptor (AR) via an AR degradation enhancer, ASC-J9^®^, or neutralization of CC-chemokine ligand (CCL) 3 with an antibody, resulted in suppression of macrophage migration and prostate stromal cell proliferation [37]. In previous research [14], the changes of androgen levels and tissue remodeling by aging are generally considered to be the main determinant factor of BPH. In prostate epithelial and stromal cells, testosterone produced by the testis spreads into the prostate epithelium and stromal cells. In stromal cells, most testosterone is changed to DHT, which can work in an autocrine manner in stromal cells or spread into nearby epithelial cells, working in a paracrine manner [38]. It has a high affinity for the AR [21,38,39], which can be regulated by the activated several growth factors or their receptors. Therefore, AR expression influences cell proliferation in both epithelial and stromal cells [40]. However, the role of androgen-independent factors in the proliferative process of BPH is also important as described above. Thus, BPH is a multifactorial disease, which further complicates the pathophysiology of BPH.

**Figure 2:**
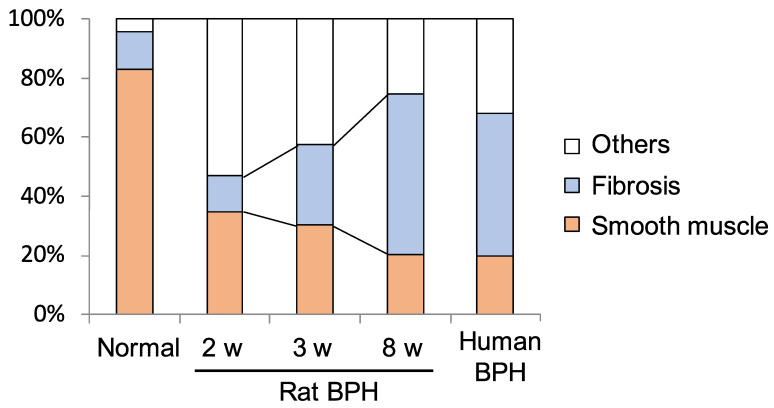
Proportion of fibrous components in rat benign prostatic hyperplasia (BPH) tissues in the BPH model at 2, 3, and 8 weeks after urogenital sinus transplantation [43].There is a time-dependent increase in the percentage of fibrous components in rat BPH tissues (normal 13.0 ± 3.9%, 2 w 12.0 ± 1.9%, 3 w 27.4 ± 3.1%, 8 w 54.2 ± 16.4%).

Several BPH rat models were developed to evaluate the stromal proliferation of BPH. Kamijo et al. reported a rat model of non-bacterial prostatitis as an animal model with the abundant stromal components [41]. However, this rat model which is produced by administering estradiol after castration has more epithelial than stromal components and was not similar to human BPH. On the other hand, Mori et al. developed the stromal-dominant BPH rat model with the urogenital sinus isolated from male rat 20-day embryos implanted into pubertal male rat ventral prostates, based on the “embryonic reawakening theory” [42]. Histological findings demonstrated that the ratio of stromal to total area was approximately 70%. This BPH rat model is characterized by the proliferation of both epithelial and stromal components with stromal-dominant, similar to human BPH tissues histologically, and it might be a suitable model for evaluating the stromal proliferation of BPH (Figure 2) [42,43]. Using this BPH model, whole-genome oligonucleotide microarray analysis of prostate specimens during the BPH proliferative process was carried out [44]. Gene ontology analysis showed that there were significant changes in gene expression associated with infection, wound healing, and the inflammatory response in BPH tissues. Functional network and pathway analyses showed that genes associated with apoptosis modulation by heat shock protein 70, interleukin (IL)-1, IL-2 and IL-5 signaling pathways, KIT signaling pathway, and secretin-like G-protein-coupled receptors class B, were relatively activated during the proliferative process in BPH tissues. In addition, the expression of genes related to the classical complement pathway and the immune response-related pathway is upregulated, suggesting the possibility that activation of the immune response is involved during the BPH proliferative process. Although these identified genes are associated with both epithelial and stromal proliferation in BPH, these may be more involved in stromal proliferation considering the histological features of this rat model.

## 5. Inflammation in BPH Proliferation

Inflammation in prostate tissues has been implicated as having the greatest role in the androgen-independent pathways in the BPH proliferative process [20,45,46,47,48,49]. In the last decades, a potentially important role of inflammation in BPH proliferation has emerged [50,51]. A relationship between inflammation and increase in prostate volume has been reported in several clinical studies [52]. The presence of inflammation is involved in increased prostate volumes and risk of acute urinary retention [46,53]. This relationship was even more pronounced when stratified by the severity of histological markers of inflammation [54]. Men with low-grade inflammation have significantly decreased prostate volume (mean prostate volume; 62 mL) compared to men with high-grade inflammation (mean prostate volume; 77 mL) [54]. These observations provide strong evidence that inflammatory cells are recruited to the prostate as a function of BPH pathogenesis, and their presence is associated with increased prostate volume. In addition to inflammatory cell proliferation, cytokines can also affect the phenotype of prostate cells. The difference in the ratio of stromal to epithelial cells in the prostate transition zone and the main inflammatory infiltrated tissue can also lead to differences in LUTS [19]. Distinguishing whether inflammation occurs in stroma or epithelial tissue is helpful for understanding the progression of BPH with immunological inflammation and LUTS. BPH patients with stromal inflammation may benefit from timely surgical treatment [55].

Chronic inflammation in BPH has been implicated in tissue remodeling through the wound healing process. Generally, injured tissues convert their biology of priority from differentiated function to a biology directed toward an expedient and effective healing process that involves an acute response, and when unresolved, chronic responses. These responses include inflammation, immune reaction, extracellular matrix remodeling, angiogenesis, and the formation of reactive stroma. Some researchers consider that tissue injury related to inflammation and subsequent chronic tissue healing processes may be an important factor in the inflammatory proliferation of BPH prostate tissues [56]. Inflammation induces cell and DNA damage, promotes cell replacement, and creates a tissue microenvironment abundance in cytokines and growth factors, thereby promoting cell proliferation and causing hyperplasia during tissue healing process [57].

Inflammatory effector molecules including cytokines, chemokines, and growth factors can activate important developmental signaling networks such as the sonic HH (SHH), insulin-like growth factor, Wnt, and Notch pathways via nuclear factor-κB and other downstream mediators of these effector molecules [58,59,60,61,62,63,64,65,66]. Histological inflammation is present in the majority of BPH specimens, providing strong evidence, with several studies showing inflammation in >98% of prostate samples [46,47,54,67]. Marberger et al. reported that the inflammatory infiltrate present in BPH consisted mostly of T cells, but B cells, macrophages, mast cells, and other cells were also present, providing robust evidence for the widespread recruitment of immune cells to BPH tissue from systemic sources [46]. Increased levels of IL-4 and IL-13 are also involved in this inflammatory infiltration [68]. Furthermore, the infiltrating T cell population is dominated by memory and suppressor phenotypes [46], consistent with repeated antigenic exposure and exhaustion during chronic inflammation over extended periods of time.

Induction of a stromal inflammatory microenvironment in the TPZ is multifactorial (Figure 1). Systemic and local ischemia during the proliferative process of BPH have also been reported to be associated with increased inflammation. In addition, the inflammation with ischemia induces various inflammatory cells, and macrophages and neutrophils were reported to provide a source of free radicals that can induce hyperplastic transformations to tissue and DNA [69]. BPH is associated with metabolic abnormality including metabolic syndrome, obesity, dyslipidemia, and diabetes mellitus. These abnormalities lead to local inflammation and elevated systemic levels of adipokines and pro-inflammatory cytokines, including adiponectin, leptin, tumor necrosis factor (TNF), IL-6, and CCL2. The bacterial or viral infections can also transform the prostate into a proinflammatory state. The presence of these stimulating factors injures prostate cells, leading to chronic inflammation [52]. For example, exogenous agents such as foreign microorganisms could produce prostatitis. Kramer et al. hypothesized the importance of the immune response in the proliferative process of BPH [46]. This study reported that the causes of immune reactions included cellular injury by infection, exposure of autoantigens due to changes in the hormonal environment, and mechanical stimulation. In particular, autoimmune reactions triggered by the exposure of autoantigens play a central role in the proliferative process of BPH.

The effectiveness of the phosphodiesterase type 5 (PDE5) inhibitor as a potential treatment for targeting inflammation in prostatic tissues has been reported. Single or repeated dosing with tadalafil, a PDE5 inhibitor, improves prostate hypoxia in spontaneously hypertensive rats [70]. In addition, Vignozzi et al. performed immunohistochemical analysis of the patients undergoing surgical treatment for BPH, and reported the inhibitory effect of vardenafil, another PDE5 inhibitor, on prostatic inflammation by CD45 immunostaining [71].

## 6. Ischemia in BPH Proliferation

Some researchers believe that prostatic ischemia may affect the BPH proliferation, leading to LUTS. Saito et al. performed an analysis using a spontaneously-hypertensive-rat (SHR rat), and found that SHR rats have significant increases in blood pressure, tissue levels of malondialdehyde/HIF-1α (as markers of hypoxia), TGF-β1/bFGF (as markers of stromal proliferation), and a significant decrease in the prostatic blood flow [72]. Furthermore, these changes were inhibited by the administration of nicorandil as antihypertensive agent, suggesting that hypertension works to decrease prostatic blood flow and promote stromal proliferation. On the other hand, one study reported the relationships between prostate size and the degree of chronic inflammation induced by local arteriosclerosis by examining the prostatic arteries removed during robot-assisted radical prostatectomy [73]. This study showed that chronic inflammation owing to local arteriosclerosis of the prostatic arteries was significantly related to prostatic enlargement through macrophage infiltration. However, most studies of the proliferation of epithelial and stromal components in BPH caused by ischemia are relative evaluations, and the degree of contribution of ischemia alone to epithelial proliferation is unknown.

PDE5 inhibitors have been reported as treatments targeting ischemia in BPH. Fujii et al. analyzed the therapeutic effect of tadarafil as a PDE5 inhibitor in chronic pelvic ischemia model rats created by iliac arterial endothelial injury and a high cholesterol diet. As a result, the chronic pelvic ischemia model rats had a decreased of prostatic blood flow, increased prostate weight, and increased prostatic stromal area, and it was reported that these changes were inhibited by administration of tadalafil [74].

## 7. Oxidative Stress in BPH Proliferation

The involvement of age-related organ inflammation and oxidative stress in the proliferative process of BPH has also been reported. Generally, aging is closely associated with oxidative stress. The oxidative stress by aging is based on the hypothesis that age-associated functional losses are due to the accumulation of reactive oxygen and nitrogen species-induced damages [75]. Furthermore, Inflammation by aging is associated with production of reactive oxygen species in the cell or tissue, which can lead to oxidation and damage of cellular components, increased inflammation [76]. Based on these reports, it is considered that oxidative stress plays a certain role in the proliferation of BPH associated with chronic inflammation of the prostate.

The association between nitric oxide synthase (NOS) and BPH have been reported in a study using animal models. Vital P et al. created transgenic mice with prostate specific expression of NADPH oxidase 4 (Nox4), which promotes the formation of NOS, and analyzed the effect of Nox4 [77]. These results showed that Nox4 expressing mice had increased oxidative DNA damage in the prostate, increased prostate weight, histological changes including epithelial, and stromal proliferation and fibrosis compared to wild type, suggesting that oxidative stress played an important role in epithelial and stromal proliferation of BPH. On the other hand, several groups have reported studies using plasma or urine from BPH patients, and these studies have showed the possibility that oxidative stress was associated with BPH proliferation [78,79,80,81].

## 8. Metabolic Syndrome in BPH Proliferation

Some studies suggested that metabolic syndrome and associated chronic inflammation also play a role in the proliferation of BPH [82]. A longitudinal study reported that increase in body mass index corresponds to an increase in prostate volume. In addition, obese participants had a 3.5-fold increased risk of prostate enlargement compared to non-obese participants [83].

Insulin is also reported to have cell proliferation promoting activity [84]. A previous study has shown that hyperinsulinemia can promote cell proliferation with prostate enlargement [85]. Insulin-like growth factor-1 (IGF-1) has been shown to have a strong mitogenic and antiapoptotic effect on prostate tissues [86]. In addition, insulin-like growth factor binding protein-3 (IGFBP-3) was found to decrease the level of IGF-1 and the antiapoptotic properties of IGF-1 via the regulating the interaction between IGF-1 and its receptor [87,88]. On the other hand, the relationship between metabolic syndrome and ischemia was also reported. Chen et al. analyzed the effect of metabolic parameters for prostatic proliferation using a rat with fructose administration [89]. In this study, the rat with fructose showed significant increases in body weight, blood pressure, plasma glucose, insulin, and triglyceride levels with a decrease in blood volume and prostate weight. In addition, histological findings of the prostate showed glandular hyperplasia, while morphometry showed increased stromal component in the rat with fructose, suggesting that BPH proliferation by metabolic syndrome was involved in both epithelial and stromal proliferation.

Recently, a treatment for BPH has been reported by targeting dyslipidemia, one of the components of metabolic syndrome [90]. This analysis using human prostate tissues and cell lines revealed that simvastatin, one of the widely used statins for dyslipidemia, could modulate cell proliferation, apoptosis, tissue fibrosis, and the EMT process in the prostate through crosstalk between peroxisome-proliferator-activated receptor gamma and WNT/β-catenin pathways.

## 9. Infection in BPH Proliferation

A possible cause of inflammation in BPH is the effect of infection in the prostate. It has long been reported that microbial, viral, and fungal infections are involved in the process of BPH proliferation. Direct involvements of *Staphylococcus*, *Acinetobacter*, *Candida*, and *Trichomonas* spp., as well as viruses, as infections have been reported [91,92]. Furthermore, as described above, it has been reported that stimulation by infection in the prostatic tissues may promote autoantigen exposure of prostate cells [46,93]. An interesting in vitro study that tried to elucidate the mechanisms of BPH proliferation by *Trichomonas vaginalis* has been reported [94]. This analysis showed that BPH-1 cells, prostate epithelial cells, incubated with live *Trichomonas vaginalis* produced CCL2, IL-1β, IL-6, and CXC motif chemokine ligand 8, and induced and activated mast cells. Furthermore, activated mast cells promoted the proliferation of prostate stromal cells and invasive capacity through inflammatory mediators, such as β-hexosaminidase and tryptase. In addition, IL-6 produced by proliferating prostate stromal cells induced the multiplication of BPH-1 epithelial cells due to stromal-epithelial interaction [95]. These studies suggested that the infection in the prostate had the possibilities to promote both epithelial and stromal proliferation through stromal-epithelial interaction.

Recent studies indicate that the microbiome at multiple anatomic sites including the gastrointestinal tract and oral cavity can affect prostate inflammation in relation to BPH [96]. An association between gut microbiota and BPH was also reported. In the analysis using prostate tissues collected by prostate biopsies, there was a higher proportion of Firmicutes and Actinobacteria spp. in the prostatic enlargement group and a higher proportion of Bacteroidetes in the normal prostate group [97]. Recent studies have uncovered the possible association between periodontal disease and prostatic disease, suggesting new prevention and therapeutic treatments for the disease by targeting periodontal pathogens [98]. Estemalik et al. investigated the oral pathogens in patients with BPH, and they found that 9 of 10 patients with BPH presented no less than one oral pathogen in their prostatic secretion samples, such as *Porphyromonas gingivalis* and *Treponema denticola* [99]. An association between the prostate microbiota and prostate enlargement has also been reported [100,101].

Several studies have reported the treatments that target microbial infection of BPH tissues. In inflammation by bacterial infection, an antibiotic agent may be the first choice. Antibiotic therapy requires a drug to reach a certain concentration, and its success depends on the drug’s antibiotic activity and pharmacokinetic characteristics [102,103]. In the treatment of chronic inflammation, phytotherapy, including pollen extract in combination with other drugs, is also one of treatment approaches. For inflammation caused by nonbacterial infection, the improvement in diet and physical exercise in daily life could regulate the composition of intestinal flora, affect the body’s metabolism and immunity, and delay the disease progression [102].

## 10. Autoimmune Reactions in BPH Proliferation

In the proliferative process of BPH, the immune response is activated by various factors including infection, mechanical stimulation, and hormonal changes, and it is accompanied by infiltration of inflammatory cells, mainly lymphocytes [46]. Approximately 90% of prostate immune cells are T lymphocytes, and cell-mediated immunity has been known to be involved in BPH [52]. Especially, CD8+ T lymphocytes are standardly located in the periglandular area around glandular epithelium, while lymphoid aggregates that consist of B lymphocytes and parafollicular T lymphocytes are located in the fibromuscular stromal area [56]. During the immune reaction, many lymphocytes promote the release of cytokines and growth factors, which further leads to abnormal remodeling of the prostate structure characterized by tissue damage, chronic immune response, and fibromuscular growth [54,56]. The immune response in BPH may occur through increased expression of IL-17, and the autoimmune response associated with T cells may induce proliferation of both epithelial and stromal cells [104]. 

As described above, the immune response in BPH proliferation might be caused by an autoimmune reaction that responds to autoantigens in prostatic tissues [46]. An interesting study of the involvement of autoimmune reactions in the prostate was published in 1987 [105]. In that study, a mouse model involving removal of the thymus immediately after birth was created. Thymectomy inhibits central immune tolerance and produces T cells and antibodies that react to autoantigens, inducing autoimmune prostatitis. At that time, prostatic autoantigens were considered to be exposed by direct damage to the extracellular connective and periglandular tissues of the prostate by the prostatic secretion, which contains a proteolytic enzyme [106]. However, what this autoantigen associated with BPH development was not clarified. Therefore, since these reports, there have been numerous studies attempting to identify the autoantigens in the BPH proliferative process.

One study reported research to extract only peripheral blood mononuclear cells and T cells from patients with granulomatous prostatitis and react them with prostate specific antigen as an autoantigen. Then, when the CD4 and CD8-positive cells that reacted were extracted and analyzed, two human leukocyte antigen molecules were identified as epitopes, that is, antigen recognition sites [107,108]. The analysis of IgG autoantibody reactivity to Lens epithelium-derived growth factor splice variant of 75 kDa (LEDGF/p75) in patients with BPH showed that autoantibody reactivity to LEDGF/p75-overexpression cells in about 50% of patients with BPH was significantly increased [109]. Furthermore, studies using a stromal-dominant BPH model rat has been previously reported [93]. Immunoprecipitation and mass spectrometry using rat BPH tissues clarified that four molecules, Hsp90, Annexin, β-actin, and α-smooth muscle actin (SMA), were identified as autoantigens associated with BPH [93]. These interesting results regarding autoantigens might present further evidence for the occurrence of autoimmune responses in BPH.

**Figure 3:**
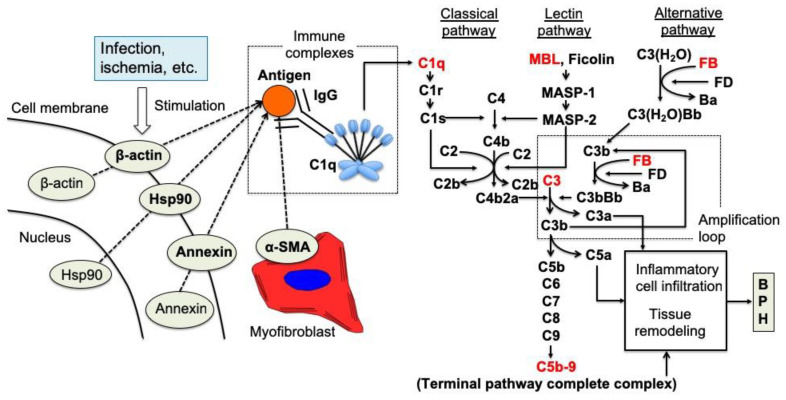
A predicted sequential model for the proliferation of BPH tissues through complement system activation in rat [93].

On the other hand, the involvement of the complement system, which is part of humoral immunity, has been reported in the proliferative process of BPH [44,93]. In the analysis using the stromal-dominant BPH model rat which has the both epithelial and stromal proliferation with time, complement components including C1q, C3, MBL, Factor B, and C5b-9 were highly expressed in rat BPH tissues compared to normal prostate tissues, and these expressions were increased time-dependently [93]. These results of complement component expression showed that the complement classical and lectin pathways were first activated in the proliferative process of BPH. The activation of complement classical and lectin pathways is reported to be involved in tissue regeneration, angiogenesis, and amplification of inflammation in addition to the well-appreciated properties of defending against foreign pathogens [110]. In addition, the complement classical pathway is known to be activated by an antigen-antibody reaction triggered by an autoantigen. From the above, autoimmune reactions might be involved in BPH proliferation including epithelial and stromal cells through the activation of the complement classical pathway (Figure 3).

Intracellular components of prostatic stromal cells, such as annexin, Hsp90, α-SMA, and β-actin, are exposed to the exterior of the cells due to infection, ischemia, or some other extracellular stimuli. Autoantibodies against the externalized components are developed, although the development mechanism is unclear, and antigen-antibody complexes are formed with the exposed intracellular autoantigens. The complement classical pathway is activated by binding of C1q to the complexes. Subsequently, activation of the lectin and alternative pathways occurs to accelerate the cleavage of C3 and C5, leading to the formation of anaphylatoxins (C3a and C5a) and the terminal pathway complex C5b-9. Our results strongly suggest that the complement system plays a role in the proliferation of BPH tissues in the rat BPH model via this complement system cascade [93].

While several studies on the involvement of autoimmune reactions in BPH proliferative process have reported, there are few reports on the treatment of BPH by targeting autoimmune reactions. However, one interesting study that analyzed the effects on the prostate in patients with systemic autoimmune diseases [111]. This analysis indicated that BPH prevalence is significantly higher among patients with autoimmune diseases. Furthermore, treating these patients with TNF-antagonists significantly decreases BPH incidence. TNF inhibition significantly is reported to decrease prostate epithelial proliferation and macrophage-mediated inflammation. Although this study was not the analysis directly targeting autoimmune reactions, it suggested that the inhibition of secondary inflammation is important for the treatment of BPH by autoimmune reactions.

## 11. Myofibroblasts as Reactive Stroma in BPH Proliferation

Generally, myofibroblasts are among the cells that constitute the stromal area of the prostate, and they also act to stimulate tissue fibrosis or stromal proliferation concurrently [112]. Myofibroblasts expresses both α-SMA and vimentin and have both smooth muscle and fibroblast functional characteristics. Myofibroblasts play a central role in fibrotic diseases of the skin, liver, pancreas, kidney, and urogenital tissues, among others. Myofibroblasts present the characteristics to secrete many growth factors and chemokines [113].

Several studies have identified the myofibroblast as an important component of the ‘reactive stroma’ associated with pre-neoplastic disease in the prostate peripheral zone [114,115]. One study showed that myofibroblasts are associated spatially with BPH glandular components that overexpress IL-8 [116] and the regulation of myofibroblast differentiation and tenascin-C expression by IL-8 [117], indicating that myofibroblasts and IL-8 were involved in the development of BPH. In other words, IL-8 is involved in myofibroblast response in stromal observed across multiple fibrotic lesions. There is also a study of myofibroblasts using the stromal-dominant BPH rat model [43]. In this BPH model, it was shown that the number of myofibroblasts in the BPH stromal area increased as BPH fibrosis progressed. In addition, the presence of cells showing the differentiation process from fibroblasts to myofibroblasts was clarified.

Some studies of the treatments targeting myofibroblasts during the proliferative process of BPH have been reported. Secreted glycoprotein Dickkopf-related protein (Dkk)-3 is altered in age-related proliferative diseases of the human prostate. Knockdown of *DKK3* significantly suppressed prostatic stromal cell proliferation, as well as fibroblast-to-myofibroblast differentiation, and also increased the expression of the vessel-stabilizing factor angiopoietin-1. Dkk-3 promotes fibroblast proliferation and myofibroblast differentiation and regulates expression of angiopoietin-1 in prostatic stroma potentially through enhancing phosphoinositide 3-kinase/protein kinase B signaling. Thus, elevated Dkk-3 in the stroma of the prostate presumably regulates stromal remodeling by enhancing proliferation and differentiation of stromal cells and by contributing to the angiogenic change observed in BPH [118]. In addition, IL-8 can induce fibroblasts in prostate stromal nodules to show significant myosin aggregation and α-SMA immune activity, suggesting that IL-8 acts as a regulator of BPH reactive stroma and is a potential therapeutic target [116].

## 12. Conclusions

BPH is a multifactorial disease, and understanding the role of androgen-independent factors contributes to elucidating the pathogenesis of BPH. Notably, the inflammation in the prostate is a key factor in the pathogenesis of BPH, and autoimmune reaction has recently attracted attention as a stimulating factor of inflammation. Several studies have identified autoantigens associated with the development of BPH, and targeted inhibition of these molecules may lead to identifying novel therapeutic targets for BPH. In addition, the complement pathway following autoimmune reactions may also be a potential therapeutic target. The field of androgen-independent factors including these immune responses has a lot of development potential, and further development of research is expected.

## Data Availability

Data sharing is not applicable to this article as no datasets were generated or analyzed during the current study.
